# Heart Rate Variability Sensing Can Reveal Characteristic Autonomic Modulation via Aromatherapy in Relation to the Effects on Feeling: A Study on Citrus Aurantium Oil and Rose Water

**DOI:** 10.3390/s25226906

**Published:** 2025-11-12

**Authors:** Toshikazu Shinba, Emi Asahi, Satoshi Sakuragawa

**Affiliations:** 1 Department of Psychiatry, Shizuoka Saiseikai General Hospital, Shizuoka 422-8527, Japan; 2 Cancer Chemotherapy Center, Shizuoka Saiseikai General Hospital, Shizuoka 422-8527, Japan; 3 Shizuoka City Industry-University Exchange Center, Shizuoka 420-0857, Japan

**Keywords:** aromatherapy, autonomic modulation, parasympathetic nerve, heart rate variability, citrus aurantium oil, rose water

## Abstract

**Highlights:**

**What is the main finding?**

- Parasympathetic activation by citrus aurantium oil was related to the changes in feeling, but that by rose water was not, indicating that the relationship between autonomic modulation via aromatherapy and subjective feeling may differ depending on the aromas.

**What is the implication of the main finding?**

- HRV sensing in relation to the effects on feeling will be a new technical advance for monitoring the effectiveness of aromatherapy.

**Abstract:**

(1) Background: There have been previous reports of autonomic modulation by aromatherapy. In this study, we recorded heart rate variability (HRV) to assess its relationship with the effects on feeling. (2) Methods: Twenty-three healthy subjects, who were blind to the aroma type, were exposed to citrus aurantium oil (CAO) or rose water (RW) aroma for 5 min using a diffuser situated in a room. Electrocardiographic data were measured continuously using a wireless device attached to the chest. R-R intervals were used to calculate HRV scores, including high-frequency (HF) variation, low-frequency (LF) variation, LF/HF ratios, the coefficient of variation in R-R (CVRR), and heart rate. A visual analog scale (VAS) was used to evaluate disfavor, fatigue, anxiety, tension, and somnolence at the end of the treatment. (3) Results: CAO significantly reduced disfavor, anxiety, and tension, while RW did not affect VAS scores. HF scores were high during the treatment with both CAO and RW, indicating parasympathetic activation. Treatment with CAO was also accompanied by an increase in LF and the CVRR, whereas treatment with RW was not. HF scores during CAO treatment were negatively correlated with somnolence. No relationships between VAS scores and HRV scores were observed in the RW treatment. (4) Conclusions: In CAO treatment, parasympathetic activation is related to feeling. RW, on the other hand, exerts its autonomic effects without changes in feeling. These results suggest that autonomic modulation by rose water may not depend on the generated feelings, suggesting the usefulness of HRV monitoring in aromatherapy.

## 1. Introduction

In addition to conventional medical treatments, natural therapies are utilized to promote health conditions [1]. They include the use of plants, wooden materials, and forest environments, and various modes of application have been developed [2,3]. Natural therapies are personalized and can be conveniently adopted upon experiencing stressful events. One such natural therapy is aromatherapy, which can take various forms, including inhalation, air diffusion, skin coating, and bathing, depending on the needs of the user. Some studies have suggested potential benefits of aromatherapy for relaxation and stress reduction, although evidence for its clinical efficacy remains limited [4,5,6,7,8].

In the obstetric field, pregnancy is often stressful even in its normal course due to changes in body condition and lifestyle, inducing anxiety. Aromatherapy was found to help pregnant women by reducing stress, including anxiety regarding labor pain [4,5,6]. In a study on patients suffering from acute coronary ischemia [7], aromatherapy was also found to reduce anxiety. A review report further indicated that insomnia could be ameliorated by aromatherapy [8]. These studies support the usefulness of aromatherapy in the medical field, in addition to the conventional treatments, including pharmacotherapy.

However, in their review report, Lee and colleagues [9] indicated that the evidence for the usefulness of aromatherapy is not sufficient for conditions such as hypertension, depression, anxiety, pain relief, and dementia. Additionally, a Cochrane database review report [10] found no convincing evidence that aromatherapy is beneficial for dementia, reporting inconsistency in the results.

The lack of consistent evidence for aromatherapy may stem from the subjectivity of evaluating the associated effects. Personal preferences pertaining to the scents may also affect the results of experiments. To address the disadvantages posed by subjective evaluation, biological mechanisms of aroma effects have been studied using physiological measures such as electroencephalography, brain blood flow, and autonomic nerve activity [11,12]. Among these measures are heart rate variability (HRV) indices, which are frequently employed to elucidate autonomic changes following administration of aromas [13,14,15]. HRV can be measured under unrestricted conditions using a wearable device or a conventional electrocardiogram machine, inducing little distress in the subjects, and is suitable for clarifying aspects of autonomic modulation in aromatherapy.

HRV is measured by analyzing the beat-to-beat intervals of heart rhythms using frequency spectrum analysis. Frequency-domain analysis reveals high-frequency variation (HF) related to parasympathetic activity and low-frequency variation (LF) related to both sympathetic and parasympathetic activity [16]. The LF/HF ratio is often used to reflect sympathetic activity. Using these autonomic measures, researchers have objectively evaluated the effects of aromatherapy in previous research.

Inhalation of citrus bergamia oil has been reported to induce relaxation, with the autonomic balance being shifted toward parasympathetic activation in healthy subjects [13]. Sympathetic and parasympathetic nervous activity decreases and increases, respectively, in dementia subjects showing agitation after several weeks of aromatherapy [14]. Rose oil significantly decreases systolic blood pressure, suggesting the lowering of autonomic arousal [15]. These data show that parasympathetic activity is enhanced following aromatherapy, leading to relaxation, although the data for various types of aromas are still insufficient.

In addition, Ikei and colleagues [17] revealed the non-aroma aspects of HRV changes during aromatherapy by finding modulations of autonomic activity after presenting subjects with images of flowers. Images are enough to induce autonomic effects. Similarly, perception of the aroma in relation to subjective feelings, including preferences and changes in mood, should influence autonomic activity, too. We previously reported the effects of depressive and anxious feelings on HRV indices [18] and suggested that feelings themselves can affect HRV.

To adequately use aroma substances and their autonomic effects in aromatherapy, it is first necessary to accumulate evidence showing the autonomic effects of various aromas. Furthermore, it is important to determine whether these effects are dependent on or independent of subjective sensation. If the effects are dependent on how the aromas are perceived, then the sensation itself is critical for realizing the optimum effects. If the effects are independent of feeling, then they can be used without personal preference concerning the choice of aroma.

In this study, two kinds of aromatic substances—citrus aurantium oil and rose water—were examined to clarify their capacity for autonomic modulation using HRV measurements in relation to changes in feeling. Both substances are widely utilized not only in aromatherapy but also in medicine, drinks, food, and social activities [19,20]. Some data regarding the autonomic modulations induced by these substances have already been reported. Using HRV measurements, Igarashi and colleagues [21] showed that rose flower induces comfortable and natural feelings and increases parasympathetic nerve activity. Rose oil also decreases blood pressure, as described above [15]. However, no convincing data are available concerning the hydrosols of rose flower—rose water. As for citrus aurantium oil, it has been reported to reduce anxiety and stress in pregnant women and patients with heart diseases [4,7]. Using blood pressure measurements and HRV, researchers found that ingestion of citrus aurantium induced early resumption of parasympathetic activities after exercise [22]. However, there is no clear evidence showing its effect on HRV.

Based on the above-mentioned background, in this study, we first examined the effects of citrus aurantium oil and rose water treatments on HRV and then analyzed the relation between their effects on the HRV indices and the changes in feeling induced by the aromas in healthy participants.

## 2. Materials and Methods

### 2.1. Experimental Rooms

**Figure 1:**
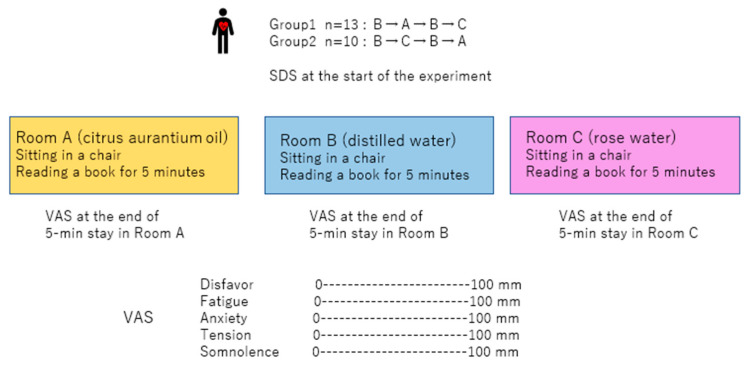
Schematic of the experimental protocol. The order of entry into the 3 rooms (A, B, and C) for the two groups (1 and 2) is presented. The self-rating depression scale (SDS) was employed at the start of the experiment. Rooms A, B, and C were filled with citrus aurantium oil, distilled water, and rose water, respectively. The participant, who was blind to the substance, sat on a chair and read a book in the room. After each stay in the room, a visual analog scale (VAS) composed of 5 lines was completed, as presented in this figure.

Three identical experimental rooms (Rooms A, B, and C) were used (width, 242 cm; length, 357 cm; and height, 250 cm). Inside each room, there was a door on one side of the wall, and a table and a chair were placed inside (Figure 1). During the experiment, the temperature was kept at 24 degrees Celsius.

### 2.2. Aromas

For aroma treatments, rose water (Rose Skin Water No. 76, Koyo Kasei Co., Ltd., Shizuoka, Japan) and citrus aurantium oil (Atami No Kaori, Peace Mind Co., Ltd., Atami, Japan) were used. They were obtained through the steam distillation of rose petals and bitter-orange pericarps, respectively, under reduced pressure at a low temperature (40 and 50 degrees Celsius for rose water and citrus aurantium oil, respectively); rose water was the hydrosol, and citrus aurantium oil was the supernatant. The citrus aurantium oil was emulsified and diluted to 0.1% in water. Distilled water was used as a control. An aroma diffuser (AD-SD2, Ryohin Keikaku Co., Ltd., Tokyo, Japan) was placed on the table in each experimental room (Rooms A, B, and C), as described above. The diffusers in Rooms A, B, and C contained citrus aurantium oil emulsified in water, distilled water, and rose water, respectively, and were switched 30 min before the start of the experiment. The ultrasonic vibration frequency was set to 2.4 MHz.

### 2.3. Participants

Thirty participants, who were members of the affiliated hospital and its associated organizations, were recruited. They were healthy and were not being treated for cardiovascular, endocrinological, neurological, and psychiatric disorders. After we explained the experimental procedures, they gave written informed consent to participate in this study. The protocol for this study was approved by the Institutional Review Board of Shizuoka Saiseika General Hospital (No. 1-17-02; approved on 18 December 2019) and conformed to the provisions of the Declaration of Helsinki.

The experiments were conducted in the evening (16:00–19:00 before supper) on 27–30 January 2020. Each participant participated in the experiment on one of these 4 days. On the day of the experiment, each participant was instructed to stay in one of the experimental rooms 4 times: once in Room A, twice in Room B, and once in Room C. The duration of each stay was 5 min. The order of the stay was as follows: B to A to B to C (Group 1), or B to C to B to A (Group 2). The participants were randomly assigned to either group to eliminate the effects of the order. The participants were excluded from analysis if they drank alcohol on the night before and on the day of the experiment or drank coffee or smoked on the day of the experiment. After exclusion, the data pertaining to 23 participants (mean age, 40.7 years; age range, 23–61; gender distribution, 9 men and 14 women) were analyzed. There were 13 (6 men and 7 women) and 10 (3 men and 7 women) participants in Groups 1 and 2, respectively.

Each participant stayed alone in each room for 5 min, sitting on a chair in front of the table where the diffuser was situated, and was instructed to read a book. The participants were not informed of the substance inside the diffuser (citrus aurantium oil, rosewater, or distilled water). A 2 min transfer and washout period outside the experimental room was implemented before the participants entered the next room. During this period, the participants were instructed to report the content of the book to the experimenter, confirming that they maintained arousal during the experiment, and complete the visual analog scale (VAS) to determine the feelings—including disfavor, fatigue, anxiety, tension, and somnolence during—they experienced during their stay (Figure 1). Disfavor corresponds to an unfavorable feeling regarding the air in the room. In addition, a questionnaire, the Self-rating Depression Scale (SDS), was completed at the start of the experiment to assess the mental state of each participant [23].

### 2.4. Heart Rate and Heart Rate Variability Measurement

We used a wearable wireless device attached to the chest of each subject throughout the experiment to measure heart rate (HR) and HRV for recording electrocardiogram (RF-ECG2, GM3 Co., Ltd., Tokyo, Japan) data. The recorded data were stored in a computer, which was placed on the table in the room and moved to the next room along with the participant. An R-R interval trend graph was resampled at the mean heart rate and used for power spectrum analysis via the maximum entropy method (MEM-Calc, GMS Co., Ltd., Tokyo, Japan). The maximum entropy method was used for HRV measurements because of its potency in analyzing data over a short period. It has been verified that a period as short as 30 s is sufficient for the analysis, in comparison with the more than 5 min period needed in the fast Fourier transform. It was recommended to use the maximum entropy method in this study because the measurement period was less than 5 min [24].

Integrated areas of power spectrum in the frequency ranges of 0.01–0.15 Hz and 0.15–0.4 Hz correspond to low-frequency (LF) and high-frequency (HF) scores for HRV, respectively [25]. The former is the heart rate variation related to blood pressure changes; it is influenced by both sympathetic and parasympathetic activity [26]. The latter is the variation related to breathing rhythms; it is influenced by parasympathetic activity [16]. We also calculated the LF-to-HF ratio (LF/HF), which was used to reflect sympathetic activity in a previous study, although this interpretation was questioned [26]. The coefficient of variation in the R-R interval (CVRR) was calculated as the ratio (%) of the standard deviation to the mean R-R interval. Instantaneous HR was also calculated for each beat-to-beat interval. These HRV and HR parameters were obtained every 2 s successively using the R-R intervals during the preceding 30 s period. The data were averaged during the period ranging from 30 s after room entry to the end of the 5 min stay to exclude the influence of heart rate variations before room entry.

### 2.5. Statistical Analysis

The data obtained during the stay in Room A (citrus aurantium) were compared with those obtained during the stay in Room B (distilled water) before entry into Room A to analyze the effects of citrus aurantium oil. The data for Room C (rose water) were compared with those for Room B (distilled water) before entry into Room C to analyze the effects of rose water (Figure 1).

The differences between the averaged data for Rooms A or C and those for Room B were analyzed using a non-parametric Wilcoxon test to reveal the effects of aroma on HR and HRV [27]. The relationships between the VAS data and the HR or HRV data were examined using Spearman’s correlation coefficients (Prism8, Version8.4.3, GraphPadSoftware, SanDiego, CA, USA) [28]. Non-parametric statistics were employed because most of the data did not show a normal distribution (Table 1).

## 3. Results

### 3.1. HRV Sensing Revealed Characteristic Autonomic Modulations Induced by the Citrus Aurantium Oil and Rose Water Treatments

**Figure 2:**
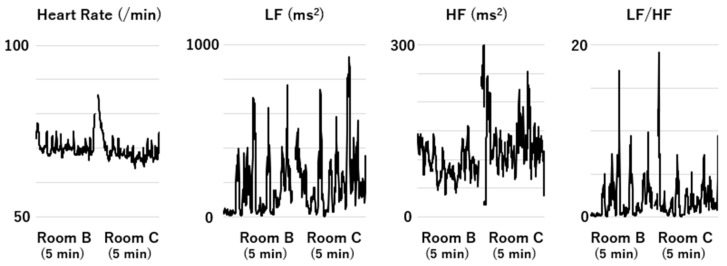
Heart rate (HR) and heart rate variability (HRV) data for a participant during their stay in Room B (distilled water) followed by those obtained during their stay in Room C (rose water). A two-minute transfer and washout period took place between these two stays. The data on the HRV and HR parameters for the period from 30 s after entry to the end of the stay were averaged for each stay to avoid the effects of the stay outside the experimental room. HR is expressed as heartbeats per minute (/min).

The HR and HRV data pertaining to a participant staying in Room B (distilled water), followed by Room C (rose water), are presented in Figure 2. The data were averaged for the period ranging from 30 s after entry into the room to the end of the stay. The means and interquartile ranges of HR, CVRR, HF, LF, and LF/HF for all the participants are presented in Table 2. The P values in Table 2 indicate that the difference between the data obtained during the stay in Room A and those obtained during the preceding stay in Room B was significant for CVRR, LF, and HF, reflecting the effects of citrus aurantium on autonomic activity. The difference between the data obtained during the stay in Room C and those obtained during the preceding stay in Room B was significant for HF, indicating the effects of rose water. HR was not altered by either scent.

### 3.2. The Different Effects of Citrus Aurantium Oil and Rose Water Treatments on VAS Scores

Table 3 shows the data for five VAS indices (disfavor, fatigue, anxiety, tension, and somnolence); the means and interquartile ranges are presented. The differences between the VAS score after the stay in Room A and that after the preceding stay in Room B were significant for disfavor, anxiety, and tension, indicating that these parameters were reduced by the citrus aurantium oil treatment. In contrast, for the rose water treatment, no differences between the VAS score after the stay in Room C and that after the subsequent stay in Room B were observed.

### 3.3. Different Relationships Between Autonomic Modulation and VAS/SDS Scores in Citrus Aurantium Oil and Rose Water Treatments

**Figure 3:**
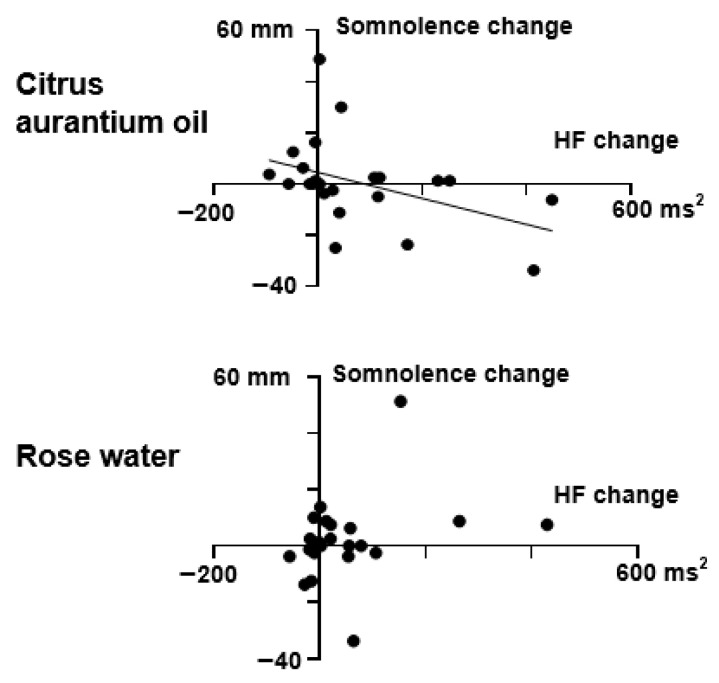
Correlation between changes in somnolence VAS scores and HF after citrus aurantium oil/rose water treatment. The changes were calculated by subtracting each subject’s score during their stay in Room B (distilled water) from that during their stay in Room A (Citrus aurantium oil) or in Room C (rose water). As for the score in Room B, that preceding the aroma exposure was used for subtraction. Filled circles represent individual data. Correlations were examined using Spearman’s correlation coefficients. A significant correlation was found for citrus aurantium oil, but not for the rose water treatment. A linear regression line is presented for the citrus aurantium oil data (r = −0.431, *p* = 0.040).

**Figure 4:**
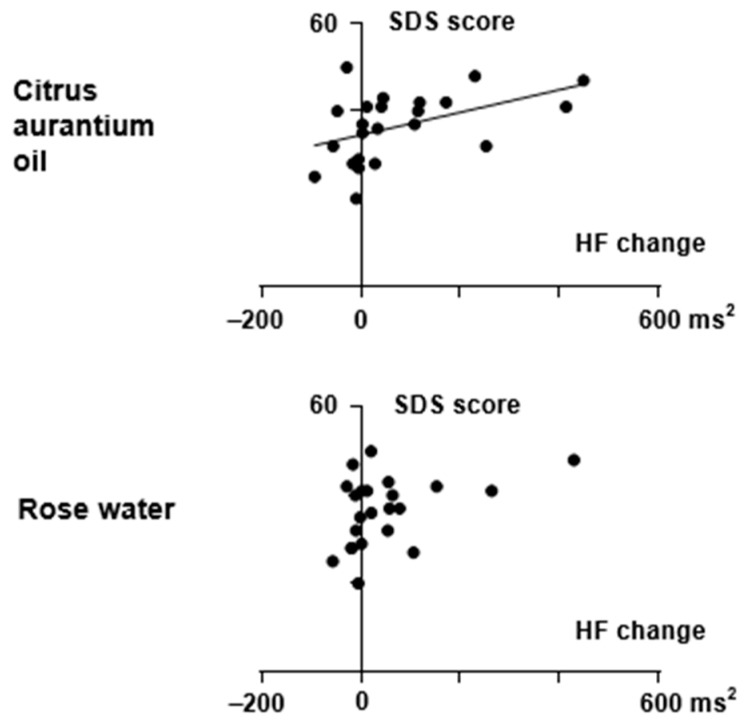
Relationship between SDS scores at the start of the experiment and changes in HF after the citrus aurantium oil/rose water treatment. The changes were calculated by subtracting the score during the stay in Room B (distilled water) from that during the stay in Room A (Citrus aurantium oil) or in Room C (Rose water) for each subject. As for the score in Room B, that preceding the aroma exposure was used for subtraction. Filled circles represent individual data. Correlations were examined using Spearman’s correlation coefficients. A significant correlation was found between two scores for the citrus aurantium oil treatment, but not for the rose water treatment. A linear regression line is presented for the citrus aurantium oil data (r = 0.537, *p* = 0.008).

As for the relationship between the VAS changes and HR or HRV changes, the change in the somnolence VAS score from the Room B measurement (distilled water) to the Room A measurement (citrus aurantium) was negatively correlated with the change in the HF score. Increases in HF scores after citrus aurantium oil treatment were correlated with decreases in somnolence (r = −0.431, *p* = 0.040, Figure 3). The change in HF score after citrus aurantium oil treatment was also positively correlated with the SDS score measured at the start of the experiment (r = 0.537, *p* = 0.008, Figure 4). These relationships were not observed in the data for rose water (r = 0.346, *p* = 0.106). Analysis of CVRR, LF, and HR data did not reveal a relationship between autonomic changes and feelings for either citrus aurantium oil or rose water (*p* > 0.05).

**Figure 5:**
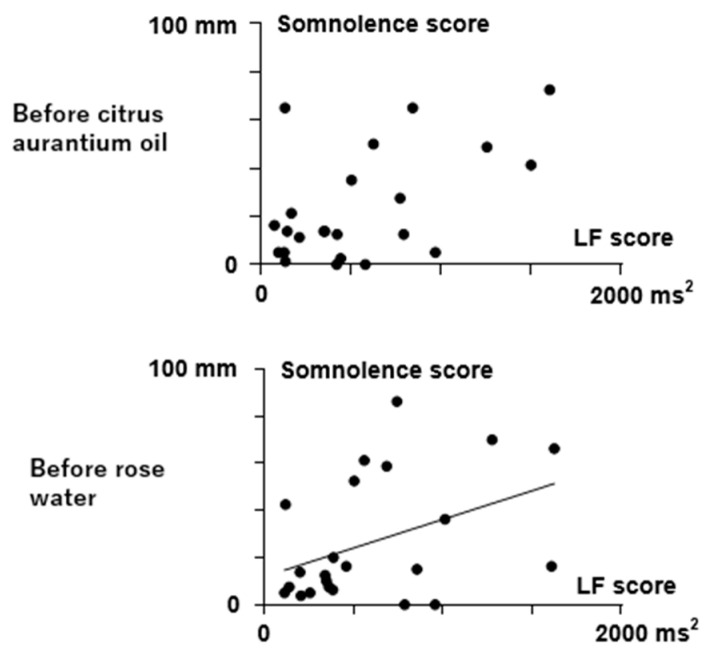
Relationship between somnolence VAS scores and LF in Room B (distilled water) before the citrus aurantium oil and rose water treatments. Filled circles represent individual data. A correlation was found between the somnolence VAS score and LF in Room B before the rose water treatment (r = 0.426, *p* = 0.043). No correlations were found during the rose water treatment (r = 0.357, *p* = 0.094).

Regarding the baseline relationship between feelings and HRV or HR scores during the stay in Room B (distilled water), a positive relationship was found between the somnolence VAS scores and LF scores before rose water diffusion (r = 0.426, *p* = 0.043, Figure 5). The relationship between somnolence VAS scores and LF scores before citrus aurantium oil diffusion was not significant (r = 0.357, *p* = 0.094). No other relationships between feelings and HRV or HR scores were observed under the control conditions during distilled water diffusion (*p* > 0.05).

## 4. Discussion

This report is the first to show that citrus aurantium oil and rose water have significant effects on autonomic activity, inducing an increase in parasympathetic activity, as found via HRV measurement. As for citrus aurantium oil, a previous study showed an early resumption of parasympathetic activity after exercise based on HRV measurements [22]. Our study directly reveals changes in parasympathetic activity indicated by an increase in HF scores. Rose water also significantly activated parasympathetic activity, as shown through an increase in HF scores. These data add evidence supporting the widely reported ability of aromatherapy to trigger parasympathetic activation and suggest that aromatherapy exerts psychological effects by modulating autonomic nerve activity. Possible neural mechanisms of the relationship between autonomic modulation and feeling could be due to their interaction at the hypothalamus and limbic cortex [29], where arousal and emotion are regulated. Aromas could affect feelings through these neuronal systems, being reflected in HF.

This study further revealed that the effects on autonomic activity can be different among the aroma substances. In this study, rose water caused changes in HF, indicating its augmenting effects on parasympathetic nerve activity. On the other hand, citrus aurantium oil produced not only an increase in HF but also an increase in LF and CVRR. The latter aroma had a wider range of HRV modulation. LF is related to both parasympathetic and sympathetic activity, and the changes in the sympathetic nerve system cannot be speculated from the results of this study.

Important differences between citrus aurantium oil and rose water were found in this study regarding the relation to feelings induced by the aromatherapy. The increase in HF score during citrus aurantium oil diffusion is significantly correlated with somnolence reduction revealed by VAS score. HF during citrus aurantium oil diffusion was the only autonomic score that manifested the relation with the changes in feeling. HRV and HR scores during rose water diffusion showed no relation with subjective sensation. Although the parasympathetic augmentation reflected in HF increment may be a common feature of aromatherapy, the dependence of the autonomic effects on the subjective sensation may be different among aroma types. It is interesting to note that citrus aurantium oil manifests its autonomic effects depending on the feeling, and that rose water acts on autonomic activity independent of feeling. The autonomic effects of rose water may not have to accompany the subjective changes to be effective in aromatherapy. It can be speculated that the substance contained in the Citrus aurantium oil, which is responsible for the autonomic effects, also causes changes in somnolence. However, the substance in the rose water, which is responsible for the autonomic effects, is different from the substance responsible for changing somnolence. The autonomic effects independent of feeling may underlie the wide use of rose water [19]. Rose water may be suitable for individuals who are too sensitive to use the citrus aromas for enhancing parasympathetic nerve activity. The user may not have to depend on their preference for the fragrance, but could instead consider the HRV sensing data when choosing the aroma.

As for the effects of citrus aurantium oil, it is also interesting that the effects on feeling and feeling-dependent autonomic modulation are different. When the data pertaining to all the participants are analyzed, this study reveals that citrus aurantium oil reduces disfavor, anxiety, and tension but does not have effects on somnolence. However, the autonomic effect is correlated with the change in somnolence. This finding also supports the idea that the autonomic effects of aromatherapy can be independent of subjective sensation. The autonomic effects are not tied to a preference for a given aroma substance. Considering the report by Ikei et al. [17] showing that only the presentation of a visual image of flowers is enough to induce HRV changes, the effects of aromatherapy on autonomic activity should be complex, including responses to images, sensations, and direct autonomic effects.

Our results on rose water are not consistent with a previous report by Tomi et al. [30] showing that hydrosols of fresh flowers did not have observable effects on heartbeat spectra. This may be due to the difference in the flower types or the modes of application. Our study is the first to show that hydrosols are effective in terms of autonomic modulation when rose is used. Hydrosols can be obtained in greater amounts than the oil component, although the fragrance is not always satisfactory. It would be interesting to use flower-derived substances with less or no fragrance for autonomic adjustment.

It is also important to note that aromas contain heterogenous substances, including β-caryophyllene, phenylethyl acetate, and 3,5dimethoxy toluene [30]. Each substance may have different profiles regarding feelings and autonomic effects. Future studies should examine each ingredient regarding the subjective and autonomic effects.

As for clinical usefulness, our data on citrus aurantium oil may support its use in managing sleep problems, as indicated by previous research [8], because they reveal HRV-dependent somnolence modulation. HF scores could be a biological index useful for adequately applying the scent of citrus in addressing sleep problems. Komori et al. previously reported that the necessary doses of antidepressants for a group of individuals were reduced after they were exposed to the fragrance of citrus [31]. Sleep problems are frequent among psychiatric patients and could be adequately modified by citrus scents with the aid of autonomic measurements. In this study, SDS scores at the start of the experiment showed correlations with the increase in HF during citrus aurantium oil diffusion. Subjects with depression scores may be able to effectively utilize citrus aurantium oil treatment to elevate parasympathetic activity. Future clinical studies are warranted to assess this issue.

Under the control conditions, during the stay in Room B (with distilled water diffusion), LF scores were correlated with somnolence before entry into Room C. No relationship was observed for HF, and the results suggest that the increase in HF in the room filled with rose water and citrus aurantium oil was not related to the baseline condition before aroma treatment. The relationship between high LF scores and high somnolence is interesting and should be studied in future research on HRV.

### Limitations

As described above, these scents contain heterogenous ingredients, and the concentrations of these ingredients may depend on the season of harvest and the location where the flowers have grown. In this study, we used scents that are commercially available and used a single lot for all participants to ensure that the treatment conditions were equal for all participants. However, further study is necessary to evaluate the effects of differences in the concentrations of ingredients to examine the aromas’ mechanisms of action. Studies on different aromas are also warranted.

As for the VASs, this study employed five parameters: disfavor, fatigue, anxiety, tension, and somnolence. More psychological parameters related to aromatherapy should be included in future research. It is also important to add other measures of autonomic activity to experiments, including skin conductance, which reflects sweating. Sweating is known to be solely controlled by the sympathetic nerves and useful for evaluating sympathetic activity [32]. Different modes of aroma presentation would also be interesting to investigate in future research, including sniffing and skin application, as they may have distinct effects on autonomic activity. Further, the multimodality of the aroma effects is an interesting area of research and should be studied regarding autonomic activity.

We admit that this study was not performed in medical patients, but the findings can be applied to patients, including those with chronic fatigue syndrome and depression, who are known to exhibit autonomic dysregulation [33]. The limitations of this study also include the gender imbalance and small sample size. Most of the HRV data did not show normal distribution (Table 1). Future studies on larger populations and with a balanced gender distribution are warranted.

In this study, we introduced a cross-over design regarding the order of aroma exposure to minimize the effects of carry-over and fatigue, and to control the blind state. We also measured HRV on one evening in a participant because measurements on separate days could have led to differences in baseline HRV levels. However, each aroma induces a characteristic sensation depending on the subject; it was difficult to completely control the blind state. Subjective evaluation, including intensity, would have affected the results. Future studies examining the influence of aroma intensity, aroma concentration, exposure duration, and exposure time of day are warranted.

As for the HRV analysis, we did not use non-linear HRV metrics in this study [34], because linear HRV is suitable for evaluating parasympathetic activity, which was the target of this study. However, future studies using non-linear metrics are warranted.

## 5. Conclusions

In this article, we propose that HRV sensing alone can be used as an objective method to monitor the effectiveness of aromatherapy, independent of the effects on feeling. Even if the aroma does not affect feeling, HRV sensing can offer feedback to aroma users regarding the autonomic effects. Regarding the continuous use of HRV sensing in real life using wearable devices, our findings highlight a technically new means of using aromatherapy to ensure parasympathetic activation, which can modulate the health state. Because parasympathetic insufficiency has been reported in somatic and mental disorders, including chronic fatigue syndrome and depression, HRV sensing will help the adequate usage of aroma in ameliorating the symptoms in these disorders.

## Figures and Tables

**Table 1:** W scores in Shapiro–Wilk test on HR and HRV data to check their normality.

	Water (Before Citrus)	Citrus	Water (Before Rose)	Rose
HR (/min)	0.9607 *	0.9341 *	0.9445 *	0.9395 *
CVRR (%)	0.9086	0.9123	0.9196	0.9240
LF (ms^2^)	0.8247	0.8226	0.8334	0.7933
HF (ms^2^)	0.7070	0.6618	0.6858	0.6819
LF/HF	0.8304	0.8786	0.9674 *	0.9368 *

* Asterisks indicate normality of data distribution. Citrus: citrus aurantium oil, Rose: rose water.

**Table 2:** The effects of citrus aurantium oil and rose water treatment on heart rate (HR) and heart rate variability scores in comparison with distilled water (CVRR, LF, HF, and LF/HF).

	**Distilled Water** **(Before Citrus Aurantium Oil)**		**Citrus Aurantium Oil**		
	**Mean**	**(95% CI)**	**Mean**	**(95% CI)**	***p***
HR (/min)	71.4	(67.4–75.3)	71.2	(66.9–75.6)	0.617
CVRR (%)	4.3	(3.6–4.9)	4.7	(3.8–5.5)	0.013
LF (ms^2^)	548.7	(356.2–741.3)	665.5	(415.7–915.2)	0.021
HF (ms^2^)	251.3	(137.8–364.9)	327.9	(163.8–492.0)	0.038
LF/HF	4.0	(2.4–5.5)	3.8	(2.6–5.1)	0.982
	**Distilled Water** **(Before Rose Water)**		**Rose Water**		
	**Mean**	**(95% CI)**	**Mean**	**(95% CI)**	**p**
HR (/min)	71.8	(67.7–75.8)	71.4	(67.3–75.4)	0.357
CVRR (%)	4.5	(3.8–5.2)	4.6	(3.9–5.4)	0.440
LF (ms^2^)	603.8	(410.6–797.0)	663.2	(416.5–909.9)	0.817
HF (ms^2^)	272.0	(145.4–398.5)	321.9	(163.1–480.8)	0.048
LF/HF	4.1	(3.1–5.2)	4.1	(2.9–5.3)	0.401

Means and 95% confidence intervals (CIs) are presented. The scores during citrus aurantium oil and rose water exposure were compared with those during distilled water exposure preceding the aroma exposure, and *p* values were calculated using the Wilcoxon test. HR is expressed as heartbeats per minute (/min).

**Table 3:** Subjective evaluations of disfavor, fatigue, anxiety, tension, and somnolence using the visual analog scale.

	Disfavor		Fatigue		Anxiety		Tension		Somnolence	
	Mean	95% CI	Mean	95% CI	Mean	95% CI	Mean	95% CI	Mean	95% CI
Water ^1^	44.8	38.4–51.2	21.3	13.8–28.8	18.9	11.9–25.8	21.4	13.1–29.7	23.4	13.6–33.3
Citrus	30.4	20.4–40.5	21.9	13.3–30.5	13.2	7.5–19.0	18.4	10.9–25.9	24.1	14.0–34.1
*p*	0.008		0.872		0.003		0.040		0.945	
Water ^2^	41.0	35.0–47.0	22.9	14.1–31.8	16.4	9.7–23.0	17.9	11.5–24.3	26.6	15.2–38.0
Rose	39.5	32.4–46.6	20.9	12.3–29.5	14.4	8.0–20.7	15.9	10.1–21.8	28.6	16.7–40.6
*p*	0.649		0.314		0.275		0.097		0.434	

Water ^1^: exposure to distilled water before citrus aurantium oil; Water ^2^: exposure to distilled water before rose water; Citrus: citrus aurantium oil; Rose: rose water; Means and 95% confidence intervals are presented; The scores during citrus aurantium oil (Citrus) and rose water (Rose) exposure were compared with those during distilled water exposure preceding the aroma exposure (Water ^1^, Water ^2^), and *p* values were calculated using the Wilcoxon test.

## Data Availability

All data for this study are available upon request from the corresponding author.
